# Spinal cord versus brain imaging biomarkers of multiple sclerosis trajectory combining 7T and 3T MRI

**DOI:** 10.1093/braincomms/fcag059

**Published:** 2026-02-25

**Authors:** Alessandro Miscioscia, Constantina A Treaba, Elena Barbuti, Valeria T Barletta, Jacob A Sloane, Eric C Klawiter, Julien Cohen-Adad, Paolo Gallo, Patrizia Pantano, Caterina Mainero

**Affiliations:** A. A. Martinos Center for Biomedical Imaging, Department of Radiology, Massachusetts General Hospital, Charlestown, MA 02129, USA; Multiple Sclerosis Clinical and Research Unit, Department of Systems Medicine, University of Rome Tor Vergata, Rome 00133, Italy; A. A. Martinos Center for Biomedical Imaging, Department of Radiology, Massachusetts General Hospital, Charlestown, MA 02129, USA; Harvard Medical School, Boston, MA 02115, USA; A. A. Martinos Center for Biomedical Imaging, Department of Radiology, Massachusetts General Hospital, Charlestown, MA 02129, USA; Department of Human Neuroscience, Sapienza University, Rome 00185, Italy; A. A. Martinos Center for Biomedical Imaging, Department of Radiology, Massachusetts General Hospital, Charlestown, MA 02129, USA; A. A. Martinos Center for Biomedical Imaging, Department of Radiology, Massachusetts General Hospital, Charlestown, MA 02129, USA; Department of Neurology, Beth Israel Deaconess Medical Center, Boston, MA 02215, USA; A. A. Martinos Center for Biomedical Imaging, Department of Radiology, Massachusetts General Hospital, Charlestown, MA 02129, USA; Department of Neurology, Massachusetts General Hospital, Boston, MA 02114, USA; NeuroPoly Lab, Institute of Biomedical Engineering, Polytechnique Montreal, Montreal, QC H3T 1N8, Canada; Department of Neuroscience, University of Padua, Padua 35128, Italy; Department of Human Neuroscience, Sapienza University, Rome 00185, Italy; IRCCS Neuromed, Pozzilli (IS) 86077, Italy; A. A. Martinos Center for Biomedical Imaging, Department of Radiology, Massachusetts General Hospital, Charlestown, MA 02129, USA; Harvard Medical School, Boston, MA 02115, USA

**Keywords:** cortical lesions, paramagnetic rim lesions, spinal cord, PIRA, 7-Tesla MRI

## Abstract

In multiple sclerosis, different types of lesions and their localization can have varying effects on clinical disability and disease progression. Ultra-high field 7-Tesla MRI improves the visualization of cortical, especially subpial, lesions and of white matter lesions with a paramagnetic rim that are associated with smoldering inflammation. Spinal cord atrophy is also a critical determinant of clinical disability in multiple sclerosis, but its importance relative to paramagnetic rim and cortical lesions in predicting neurological disability and its progression remains unclear. In this longitudinal study, we aimed to identify the most relevant predictors of both the baseline Expanded Disability Status Scale status and 4-year progression independent of relapse activity in a heterogeneous multiple sclerosis cohort. One-hundred-twelve patients (83 relapsing-remitting and 29 secondary progressive; mean age 42.3 years, mean disease duration 9.8 years) underwent 7-Tesla T2* susceptibility-weighted images to segment paramagnetic rim lesions, non-rim white matter lesions and cortical lesions; 3-Tesla T1-weighted brain MRI images extended to the C2-C3 spinal cord were employed to obtain brain volumes and the spinal cord C2-C3 cross-sectional area using FreeSurfer and Spinal Cord Toolbox. Clinical disability was assessed through the Expanded Disability Status Scale at baseline and, in 97/112 patients (86.6%), after a mean follow-up of 4.0 years. The association between imaging metrics and clinical outcome was evaluated using correlations and regression models, corrected for age, sex, treatment class and clinical follow-up time. The main predictors of baseline Expanded Disability Status Scale were cortical lesion (*β* = 2.9 × 10^−4^, *P* = 0.001), non-rim white matter lesion (*β* = 1.2 × 10^−4^, *P* < 0.001) volumes, brain white matter volume (*β* = −15.68, *P* = 0.017) and C2-C3 cross-sectional area (*β* = −0.68, *P* = 0.003). At follow-up, 23/97 patients (24%) experienced progression independent of relapse activity. Progression independent of relapse activity was associated with paramagnetic rim lesion volume (odds ratio = 1.0006 per mm³ increase, *P* = 0.030), cortical lesion volume (odds ratio = 1.0005 per mm³ increase, *P* = 0.011) and brain white matter volume (odds ratio = 0.97 × 10^−20^, *P* < 0.001). However, a stepwise logistic regression model assessing clinical, lesion and atrophy variables identified cortical lesion volume as the strongest independent predictor of progression independent of relapse activity (odds ratio = 1.0006 per mm³ increase, *P* = 0.005). In multiple sclerosis, different imaging biomarkers contribute differently to current disability and progression independent of relapse activity. Spinal cord atrophy mainly explains the current Expanded Disability Status Scale, while brain white matter atrophy and paramagnetic rim lesions provide additional insights into future disability trajectory. Among all markers, cortical lesions emerged as the main driver for progression independent of relapse activity.

## Teaser

This study defines the hierarchy of MRI biomarkers predicting progression independent of relapse activity in multiple sclerosis. Miscioscia *et al.* report cortical lesions as the strongest predictor, followed by brain white matter atrophy and paramagnetic rim lesions. Non-rim white matter lesions and cervical spinal cord atrophy primarily reflected current disability.

## Graphical Abstract

**Graphical Abstract:**
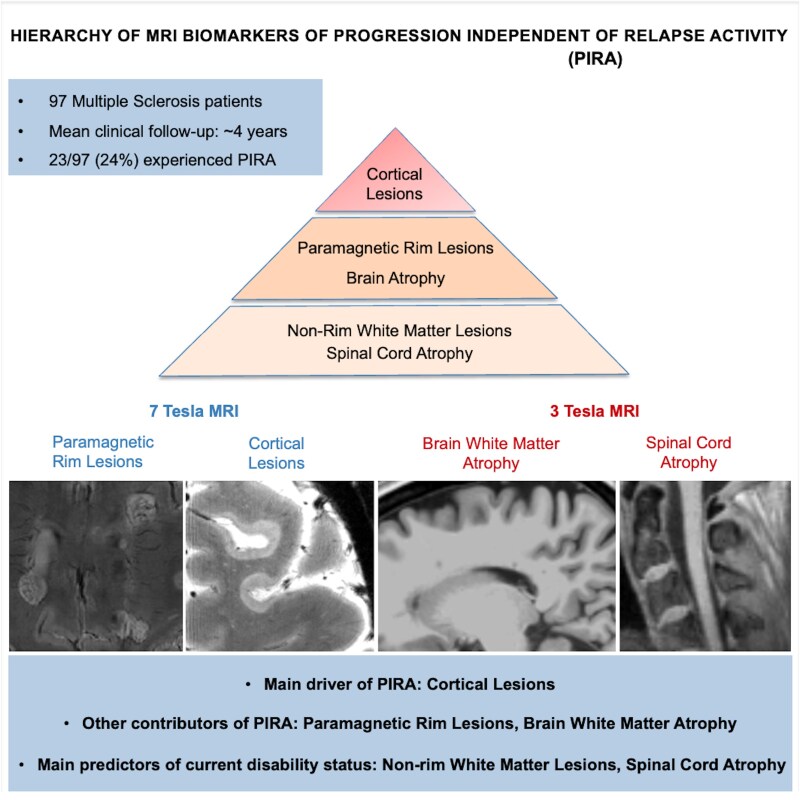

## Introduction

Multiple sclerosis (MS) is a chronic inflammatory, demyelinating and neurodegenerative disease of the central nervous system (CNS), and the most common cause of neurological disability in young adults after trauma.^1^ Disability accumulation in MS can result from either incomplete recovery following relapses (relapse-associated worsening [RAW]) or progression that occurs independently of relapse activity (PIRA).^2^ MRI biomarkers of brain and spinal cord (SC) damage can provide crucial information about the trajectory of clinical disability in MS.^3^ Particularly, paramagnetic rim lesions (PRLs), slowly expanding lesions (SELs), cortical lesions (CLs), as well as volumetric measures of SC were shown to be strong predictors of PIRA.^4-10^ In a previous 7-Tesla (7T) MRI study, we identified PRL and CL volumes as top predictors of Expanded Disability Status Scale (EDSS) progression,^6^ while SC involvement was not assessed. A previous study has shown that both SC atrophy and PRL burden are associated with PIRA,^5^ but the impact of cortical lesion load was not investigated. Although the individual contributions of SC abnormalities and CLs relative to PRLs have been previously analysed,^5-7^ to date, no study has simultaneously assessed the influence of these three biomarkers on MS disability progression. Moreover, most studies investigating brain lesion measures have been conducted using 3-Tesla (3T) MRI.^5,7^ Ultra-high field 7T MRI is considered the gold standard for the in vivo detection of CLs^11^ and PRLs,^12^ allowing for the identification of a significantly higher number of lesions^13^ compared to lower field strength and improving the differentiation among different CL types.^14,15^ Assessing lesion loads with highly sensitive methods may improve the reliability of associations with clinical outcomes and help clarify their relative contribution compared to SC atrophy in driving disability progression.

In a large single-centre MS cohort, this study evaluated brain lesion biomarkers from 7T acquisitions, including CLs and PRLs, and brain and spinal cord atrophy from 3T MRI to identify key predictors of baseline disability and the risk of PIRA over 4 years. We hypothesized that the high sensitivity of 7T MRI for smoldering biomarkers (CLs and PRLs) would enhance their prognostic value relative to other imaging brain and SC metrics.

## Materials and methods

### Standard protocol approvals, registrations, and patient consents

The study was approved by the Mass General Brigham Institutional Review Board (#2007P001274) and written informed consent in accordance with the Declaration of Helsinki was obtained from all participants before study enrolment.

### Study population

**Figure 1:**
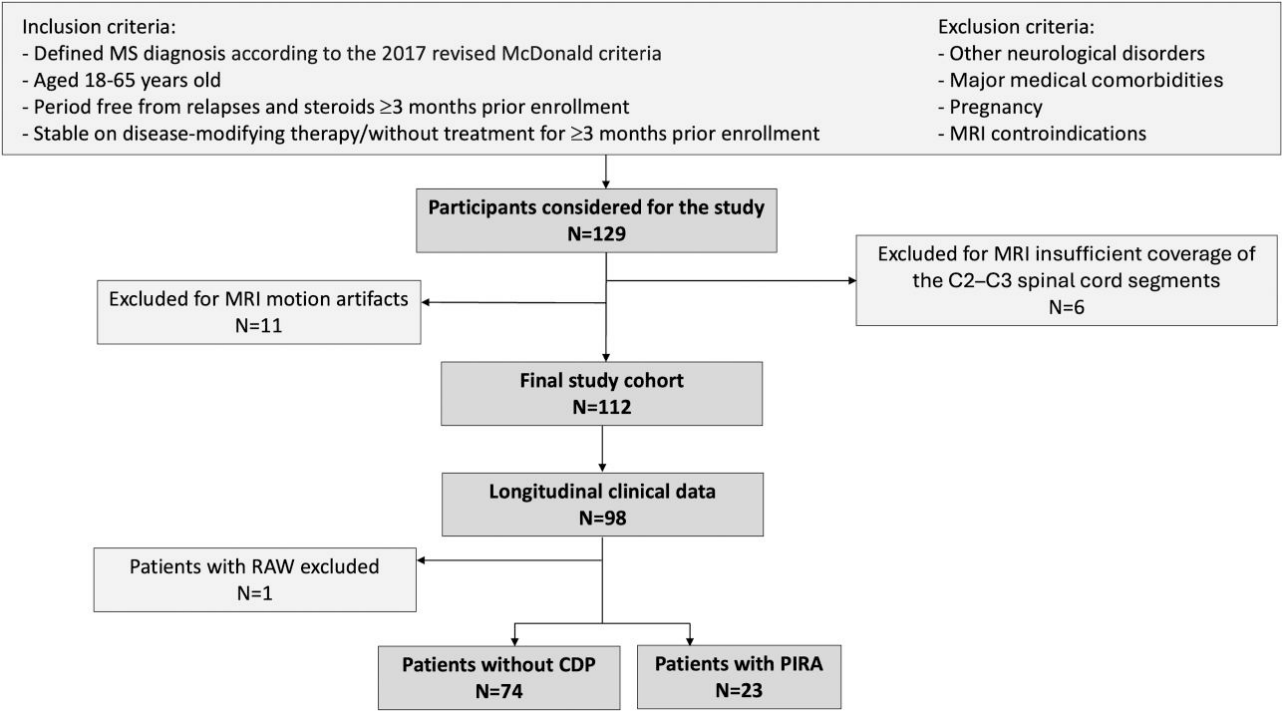
**Study flowchart.** Flowchart detailing the inclusion and exclusion criteria and the selection process of the MS study cohort. CDP = confirmed disability progression; MS = multiple sclerosis; PIRA = progression independent of relapse activity; RAW = relapse-associated worsening.

A total of 129 patients meeting MS diagnostic criteria^16^ and 41 age-matched healthy controls (HC) were prospectively enrolled in the study between 2010 and 2021. Inclusion and exclusion criteria are reported in Fig. 1. All patients were either on stable disease-modifying therapy (DMT) or without treatment for at least three months.

### Clinical assessment

All patients underwent a neurological examination within 2 weeks of the MRI scan (baseline timepoint) with neurological disability assessed using the EDSS.^17^ Neurological evaluations were regularly performed every 6–12 months during a mean follow-up period of 4.0 years (SD ± 1.9) in 98/112 patients (75 relapsing-remitting [RRMS], 23 secondary progressive [SPMS]). Confirmed disability progression (CDP) was defined as a disability increase in EDSS score, confirmed at least after 12 months, of: (1) ≥ 1.5 points if the baseline EDSS was 0, (2) ≥ 1.0 point if the baseline EDSS was between 1.0 and 5.0, and (3) ≥ 0.5 points if the baseline EDSS was ≥5.5. During the follow-up, CDP events were classified as PIRA if no relapses occurred (i) between the EDSS increase and the preceding reference visit (conducted at least 90 days earlier), and (ii) between the EDSS increase and the confirmation of disability progression.^18^ Since the study aimed to identify predictors of PIRA, patients with documented clinical relapses that resulted in disability worsening (RAW) were excluded from the longitudinal analysis. Patients were then classified as either patients who developed PIRA or patients without CDP. The DMT class in which the patient was on was classified as either none, low-moderate efficacy therapy (LMET), or high-efficacy therapy (HET).^19^

### MRI protocol

All study participants underwent two imaging sessions within a week on both a 7T and a 3T MRI human scanners (Siemens, Erlangen, Germany) using 32-channel coils. The following sequences were acquired: (i) 7T 2D fast low-angle shot (FLASH) T2*-weighted spoiled gradient-echo (GRE) image, covering the supratentorial brain; (ii) 7T T1-weighted 3D magnetization-prepared rapid acquisition gradient echo (MPRAGE) for registration purposes; and (iii) 3T T1-weighted 3D magnetization-prepared rapid acquisition with multiple gradient echoes (MEMPRAGE), covering the brain and the SC until the segment C4. The full MRI protocol is detailed in Supplementary Table 1.

### MRI data processing

#### Lesion segmentation

**Figure 2:**
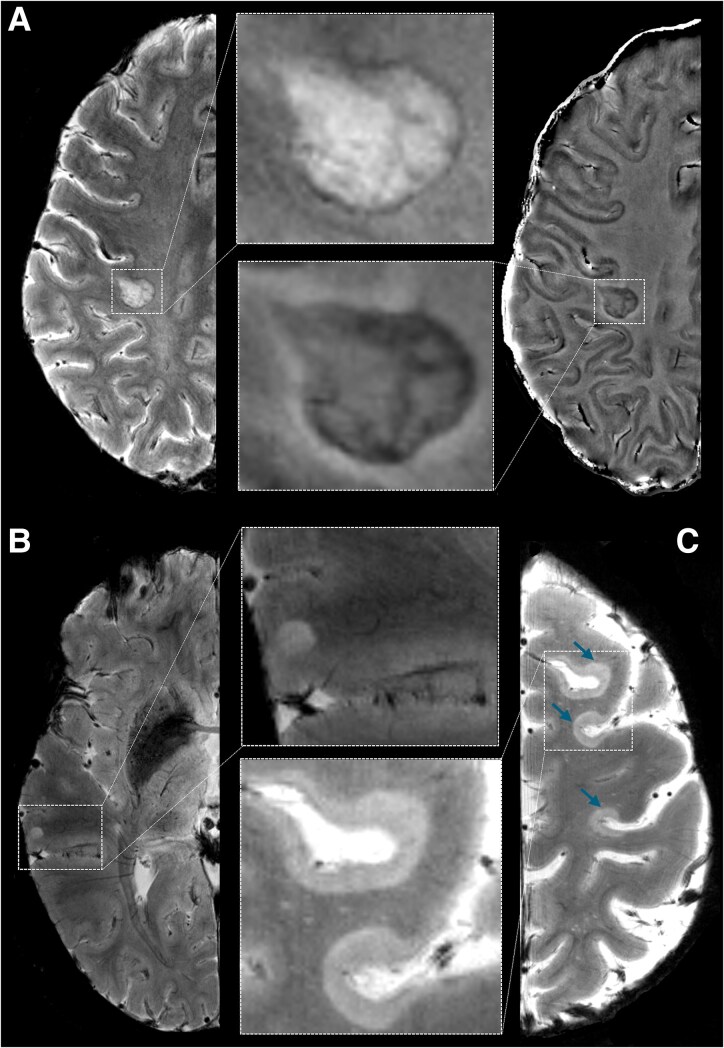
**Examples of paramagnetic rim and cortical lesions.** (**A**) shows a PRL on 7T T2* magnitude (left) and unwrapped phase (right) images, along with their respective magnifications. Examples of cortical lesions are shown on 7T T2* magnitude images, namely one leukocortical lesion (**B**) and three subpial lesions (**C, blue arrows**) also magnified. PRL = paramagnetic rim lesion.

Cortical and white matter (WM) lesions were segmented on magnitude images from 7T T2* scans by two independent raters, one radiologist (CAT) and one neurologist (CM), each with over 20 years of experience in neuroimaging analysis, followed by a consensus review, using a semi-automated tool in 3D Slicer version 4.2.0. Cortical lesions were defined as clearly hyperintense lesions within the cortex, potentially extending towards the WM, covering at least 3 voxels across two consecutive slices, including subpial, intracortical and leukocortical lesion subtypes.^15^ Paramagnetic rim lesions were defined as WM lesions having a T2-hyperintense core, surrounded by a paramagnetic rim on phase images, along at least 2/3 of the lesion perimeter, and either lacking gadolinium enhancement or present in another MRI scan occurring at least 3 months before or after the initial scan, according to recommended guidelines.^12^ Based on this criterion, WM lesions were classified as either non-rim or PRLs. Figure 2 shows examples of different brain lesion phenotypes. Lesion masks from 7T T2* were registered onto the 3T anatomical FreeSurfer reconstructions using a boundary-based registration method as previously detailed^20^ (FreeSurfer version 6.0) and lesion volumes were quantified using fslstats (FMRIB Software Library, FSL, v. 5.0, Oxford, UK).

#### Brain volume and spinal cord area measurements

**Figure 3:**
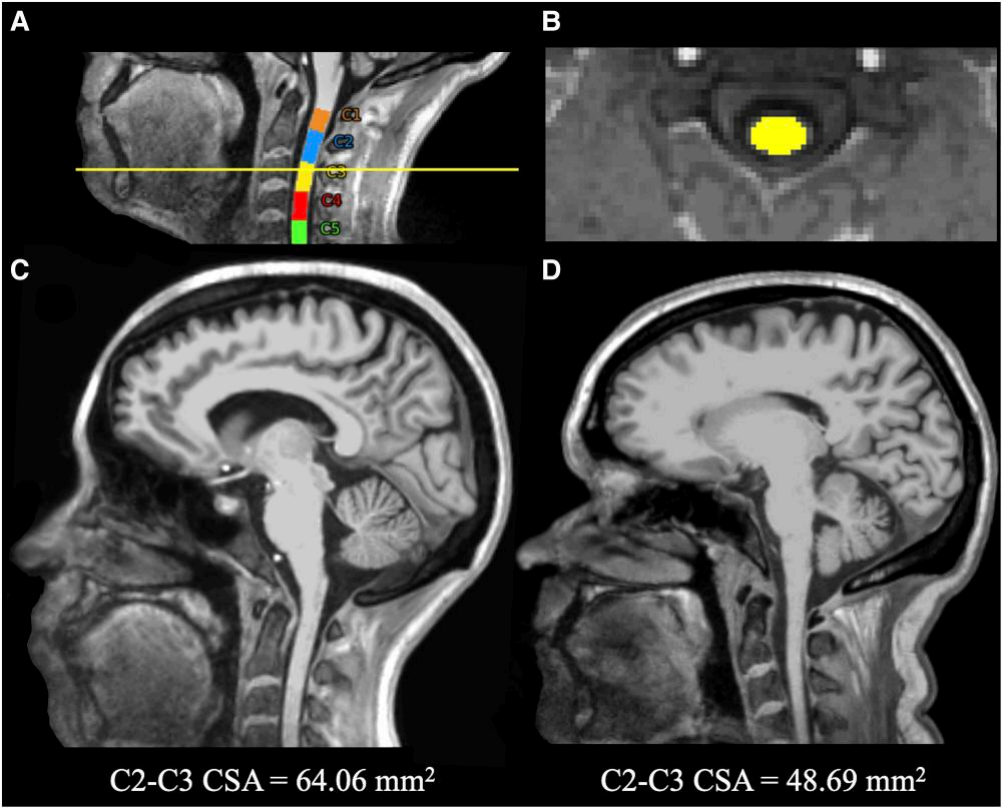
**Segmentation of cervical spinal cord and its cross-sectional area estimation.** Example of automatic segmentation of the cervical spinal cord on 3T T1-weighted sagittal (**A**) and axial (**B**) planes to estimate the C2-C3 CSA, using Spinal Cord Toolbox version 6.1. Different colours in panel **A** indicate the segmentation of individual cervical spinal cord levels, and the yellow line denotes the axial slice at the C3 level for which the spinal cord segmentation is shown in panel **B**. Sagittal T1-weighted images show the difference in C2-C3 CSA between a 29-year-old HC (**C**) and a 49-year-old SPMS patient (**D**). CSA = cross-sectional area; HC = healthy control; SPMS = secondary progressive multiple sclerosis.

Cortical thickness and brain WM volume were quantified on 3T three-dimensional T1-weighted MEMPRAGE images, after lesion filling, using FreeSurfer version 6.0. Brain WM volume was normalized by the total intracranial volume. For SC morphological analysis, we measured the mean cross-sectional area (CSA) across C2-C3 vertebral levels through the DeepSeg algorithm from the Spinal Cord Toolbox (SCT) version 6.1^21^ using 3T T1-weighted images as input. The C2-C3 intervertebral disk was manually labelled in each image to ensure optimal placement, and all pipeline steps were manually reviewed. Figure 3 shows SC C2-C3 segmentation and CSA estimation.

### Statistical analysis

Statistical analyses were performed using IBM SPSS Statistics (Version 25.0). Differences between groups (i.e., MS patients versus HC; RRMS versus SPMS patients; patients without CDP versus patients with PIRA) were analysed using the chi-squared test for categorical variables, the independent-samples *t*-test for parametric continuous variables and the Mann–Whitney test for nonparametric continuous variables. Since the occurrence of PIRA depends on the length of the follow-up interval, longitudinal analyses were thoroughly adjusted for follow-up time. We investigated the association of imaging metrics with baseline EDSS using multivariable linear regressions adjusted for age, sex, and DMT class, and with PIRA using multivariable logistic regressions adjusted for age, sex, DMT class, and clinical follow-up interval. Spearman’s rank correlations were used for exploratory analyses conducted separately in RRMS and SPMS. To determine which baseline features were independently associated with the outcome when considered together, we performed a multivariable stepwise logistic regression to predict PIRA (versus non-CDP). Collinearity between predictors in regression models was excluded based on the Variance Inflation Factor (VIF). A receiver operating characteristic (ROC) curve was used to determine the optimal cut-off volume of the predictive biomarker that best distinguishes patients at risk of PIRA from those who are not. A *P*-value of 0.05 was considered statistically significant.

## Results

### Demographic, clinical and MRI characteristics of the study population

Out of 129 MS patients, data from 11 participants were excluded due to motion artefacts, and 6 due to insufficient coverage of the C2–C3 spinal cord segments within the brain MRI field of view. A total of 112 patients were included in the final analysis. At baseline, 72 (64%) had at least one PRL, and 107 (95%) had at least one CL.

After a mean follow-up period of 4.0 (SD 1.9) years, 9 out of 98 patients (all with RRMS) experienced at least one relapse during the follow-up; however, only one of them (an RRMS patient who experienced an EDSS increase from 1.0 to 3.0, confirmed at follow-up visits) had RAW and was excluded from the longitudinal analysis. The final longitudinal cohort included 97 patients (74 RRMS, 23 SPMS), of whom 23 (24%) with PIRA (13 RRMS, 10 SPMS). Table 1 summarizes the main demographic, clinical and MRI features of HC and MS patients, categorized by clinical phenotype and longitudinal disability trajectory.

**Table 1:** Demographics, clinical and radiological characteristics

	MS	HC	MS versus HC *P*-value	RRMS	SPMS	RRMS versus SPMS *P*-value	Patients without CDP	PIRA	Patients without CDP versus PIRA *P*-value
Subjects, *n* (%)	112	41	-	83 (74%)	29 (26%)	-	74 (76%)	23 (24%)	-
Age, years, mean (SD)	42.3 (9.8)	40.0 (10.3)	0.193^a^	40.7 (9.5)	47.0 (9.3)	**0**.**003^a^**	42.7 (9.9)	41.2 (8.8)	0.516^a^
Age at onset, years, mean (SD)	32.4 (9.5)	-	-	33.7 (9.1)	28.3 (9.8)	**0**.**013^a^**	33.2 (9.6)	30.1 (8.6)	0.184^a^
Female, *n* (%)	84 (75%)	25 (61%)	0.090^b^	66 (80%)	18 (62%)	0.062^b^	57/17	19/4	0.570^b^
Disease duration, years, mean (SD)	9.8 (10.0)	-	^-^	6.8 (8.2)	18.2 (9.9)	**<0**.**001^a^**	9.2 (10.5)	11.4 (8.8)	0.367^a^
EDSS at baseline, median (IQR)	2.5 (2.0–4.0)	-	-	2.0 (1.0–2.5)	6.0 (5.0–6.5)	**<0**.**001^c^**	2.0 (1.5–3.5)	3.5 (2.0–5.5)	0.090^c^
EDSS at follow-up**^d^**, median (IQR)	2.5 (2.0–4.0)	-	-	2.0 (1.0–2.5)	6.0 (5.5–6.5)	**<0**.**001^c^**	2.5 (1.5–3.5)	5.0 (3.0–6.5)	**<0**.**001^c^**
Delta EDSS**^d^**, median (IQR)	0.4 (0.6)	-	-	0.0 (0–0.5)	0.5 (0–1.5)	**<0**.**001^c^**	0 (0–0)	1.0 (1.0–1.5)	**<0**.**001^c^**
non-CDP/PIRA, *n*	74/23	-	-	61/13	13/10	**0**.**011^b^**	-	-	-
DMT (off/LMET/HET), *n*	16/65/31	-	-	12/50/21	4/15/10	0.770^b^	13/44/17	2/14/7	0.524^b^
Non-rim WM lesion volume (mm^3^), median (IQR)	1707 (455–6161)	-	-	919 (284–3679)	5291 (2838–11 507)	**<0**.**001^c^**	1376 (325–4373)	3938 (818–8339)	0.053^c^
PRL volume (mm^3^), median (IQR)	81 (0–420)	-	-	52 (0–279)	280 (0–1035)	0.143^c^	50 (0–341)	298 (45–1796)	**0**.**015^c^**
CL volume (mm^3^), median (IQR)	436 (162–1118)	-	-	293 (122–805)	1282 (586–3348)	**<0**.**001^c^**	403 (135–971)	742 (207–3459)	**0**.**044^c^**
Brain WM volume, mean (SD)	0.294 (0.026)	0.314 (0.040)	**0.001^a^**	0.301 (0.024)	0.277 (0.026)	**<0**.**001^a^**	0.303 (0.023)	0.281 (0.089)	**<0**.**001^c^**
Cortical Thickness (mm), mean (SD)	2.37 (0.12)	2.43 (0.12)	**0.002^a^**	2.38 (0.11)	2.32 (0.12)	**0**.**005^a^**	2.37 (0.11)	2.40 (0.11)	0.355^c^
C2-C3 CSA (mm^2^), mean (SD)	58.05 (8.22)	63.16 (4.85)	**0.001^a^**	59.27 (7.71)	53.9 (8.71)	**0**.**004^a^**	58.6 (7.45)	56.0 (8.9)	0.169^a^

Significance testing:

^a^2-tailed *t* test on means.

^b^Chi-squared test.

^c^Mann–Whitney test.

^d^Clinical follow-up interval, mean (SD): 4.0 years (1.9) on 97 patients (74 RRMS, 23 SPMS) (74 non-CDP, 23 PIRA).

Bold indicates a statistically significant difference with a *P*-value < 0.05.

All brain volumes are normalized for total intracranial volume.

Abbreviations: CDP: confirmed disability progression; CL: cortical lesion; CSA: cross-sectional area; DMT: disease-modifying therapies; EDSS: Expanded Disability Status Scale; HC: healthy controls; IQR: inter-quartile range; MS: multiple sclerosis; PIRA: progression independent of relapse activity; PRL: paramagnetic rim lesion; RRMS: relapsing-remitting MS; SD: standard deviation; SPMS: secondary progressive MS; WM: white matter.

Compared to HC, patients with MS had lower normalized brain WM volume (*P* = 0.001), cortical thinning (*P* = 0.002) and reduced SC C2-C3 CSA (*P* = 0.001). Compared to RRMS, SPMS patients were older (*P* = 0.003), had longer disease duration (*P* < 0.001) and exhibited higher EDSS scores (*P* < 0.001). SPMS patients demonstrated a significantly greater lesion burden, except for PRL volume, and more pronounced volumetric reductions across all MRI metrics (*P* < 0.005 for all comparisons).

Compared to patients without CDP, patients with PIRA had significantly higher EDSS at follow-up (*P* < 0.001), higher PRL volume (*P* = 0.015), higher CL volume (*P* = 0.044), and lower brain WM volume (*P* < 0.001).

### MRI biomarkers associated with current MS disability

At baseline, linear regression models (Table 2), adjusted for age, sex, and DMT class, showed significant associations between current EDSS and volumes of non-rim WM lesions (*β* = 1.2 × 10^−4^, *P* < 0.001), CLs (*β* = 2.9 × 10^−4^, *P* = 0.001), brain WM (*β* = −15.68, *P* = 0.017) as well as SC C2-C3 CSA (*β* = −0.68, *P* = 0.003). Among covariates, age was the only one significantly associated with baseline EDSS (*β* = 0.069, *P* = 0.001). Exploratory analyses stratified by clinical phenotype demonstrated a significant positive correlation between CL volume and baseline EDSS in RRMS (*ρ* = 0.303, *P* = 0.006), whereas in SPMS only trends were observed, likely attributable to the smaller sample size (*n* = 29) (Supplementary Table 2).

**Table 2:** Regression analyses of MRI biomarkers with baseline EDSS and PIRA

Multivariable regressions							
	Baseline EDSS^a^			PIRA^b^			
MRI variables	R^2^	Unstandardized *β* (95% CI)	*P*-value	AUC	OR (95% CI)	β	*P*-value
Non-rim WM lesion volume	0.247	1.2 × 10^−4^ (0.6 × 10^−4–^1.8 × 10^−4^)	**<0**.**001**	0.631	1.0001 (0.9999–1.0002)	1.0 × 10^−4^	0.052
PRL volume	0.181	1.2 × 10^−4^ (0.3 ×10^−4–^2.7 ×10^−4^)	0.113	0.658	1.0006 (1.0001–1.0010)	5.7 ×10^−4^	**0**.**030**
CL volume	0.238	2.9 × 10^−4^ (1.2 × 10^−4–^4.6 × 10^−4^)	**0**.**001**	0.633	1.0005 (1.0001–1.0009)	5.3 × 10^−4^	**0**.**011**
Brain WM volume	0.198	−15.68 (−28.61—−2.76)	**0**.**017**	0.238	0.97 × 10^−20^ (0.10 × 10^−30–^0.95 × 10^−9^)	−46.07	**<0**.**001**
Cortical Thickness	0.179	−2.72 (−5.91–0.46)	0.094	0.569	0.991 (0.121–1.825)	−2.86	0.260
C2-C3 CSA	0.241	−0.68 (−0.11—−0.02)	**0**.**003**	0.426	0.946 (0.883–1.013)	−0.056	0.113
Age	-	0.069 (0.030–0.107)	**0**.**001**	-	0.979 (0.939–1.031)	−0.21	0.428
Sex	-	−0.077 (−0.928–0.773)	0.859	-	0.608 (0.164–2.259)	−0.497	0.458
DMT class	-	−0.703 (−1.548–0.142)	0.103	-	1.437 (0.622–3.323)	0.343	0.397
follow-up duration	-	-	**-**	-	1.416 (1.060–1.892)	0.348	**0.019**

Stepwise forward logistic regression for predicting PIRA^c^							
	MRI variables retained	Nagelkerke *R*²	OR (95% CI)		β		*P*-value
**1st step**	CL volume	0.200	1.0006 (1.0002–1.0010)		0.006		**0.005**
**2nd step**	Brain WM volume	0.298	0.89 × 10⁻^15^ (0.43 × 10⁻²⁷—0.002)		−34.6		**0.017**
	CL volume		1.0004 (1.0002–1.008)		4.5 × 10^−4^		**0.038**

^a^Multivariable linear regressions including the single MRI biomarker along with age, sex, and DMT class as independent variables.

^b^Multivariable logistic regressions including the single MRI biomarker along with age, sex, DMT class, and follow-up duration as independent variables.

^c^Stepwise forward logistic regression including age, sex, DMT class, follow-up duration, disease duration, clinical phenotype, non-rim WM lesion volume, PRL volume, CL volume, WM volume, cortical thickness, and C2–C3 CSA as independent variables.

Bold indicates a statistically significant difference with a *P*-value < 0.05.

Abbreviations: CI: confidence interval; CL: cortical lesion; CSA: cross-sectional area; DMT: disease-modifying therapies; EDSS: Expanded Disability Status Scale; OR = odds ratio; PIRA: Progression independent of relapse activity; PRL: paramagnetic rim lesion; WM: white matter.

### MRI predictors of PIRA

ogistic regression models (Table 2), adjusted for age, sex, DMT class, and clinical follow-up time, identified associations between PIRA and PRL volume (odds ratio [OR] = 1.0006 per mm³ increase, 95% confidence interval [CI] 1.0001–1.0010, *P* = 0.030), CL volume (OR = 1.0005 per mm³ increase, 95% CI 1.0001–1.0009, *P* = 0.011) and normalized brain WM volume (OR = 0.97 × 10^−20^, 95% CI 0.10 × 10^−30^–0.95 × 10^−9^, *P* < 0.001). Among covariates, follow-up duration was the only factor associated with PIRA (OR = 1.416, 95% CI 1.060–1.892, *P* = 0.019). Exploratory analyses stratified by clinical phenotype, although limited by smaller subgroup sizes, indicated that in RRMS, PIRA remained significantly associated with lower normalized brain WM volume (*ρ* = −0.317, *P* = 0.006), whereas in SPMS, PIRA correlated with higher CL volume (*ρ* = 0.502, *P* = 0.015) (Supplementary Table 2).

**Figure 4:**
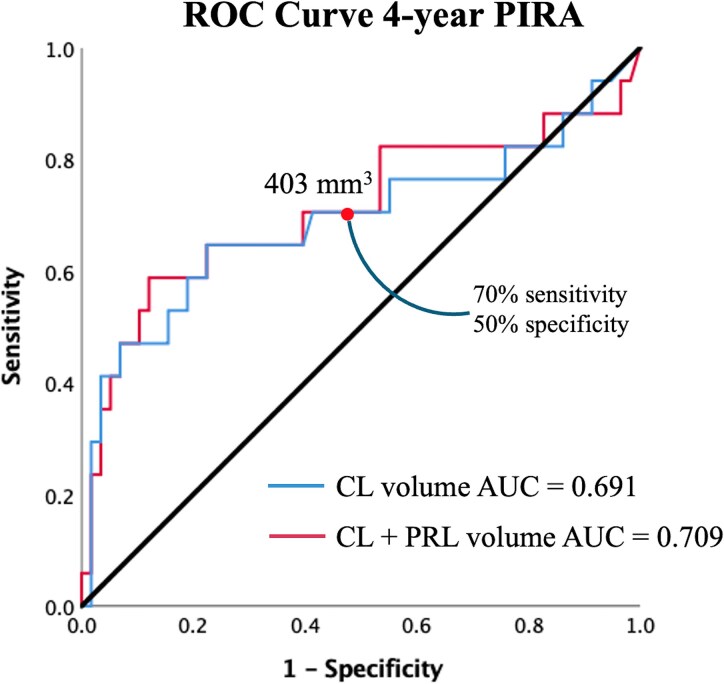
**Risk of PIRA explained by baseline cortical lesion volume alone and in combination with PRL volume.** Since CL volume was found to be the best independent predictor of PIRA, a ROC curve was plotted to quantify the CL load associated with this increased risk. Each point of the ROC curve represents the sensitivity and false positive rate (1—specificity) obtained by applying different cut-off values of baseline CL volume alone (blue line) or combined with PRL volume (red line) to discriminate patients who developed PIRA from those who did not. Adding PRL volume to CL volume resulted in a modest improvement in predictive accuracy. AUC = area under the curve; CL = cortical lesion; PIRA = progression independent of relapse activity; PRL = paramagnetic rim lesion; ROC = receiver operating characteristic.

LTo further explore independent predictors of PIRA in the whole cohort, a stepwise forward logistic regression analysis was conducted, including age, sex, DMT class, follow-up duration, disease duration, clinical phenotype (RRMS/SPMS), non-rim WM lesion volume, PRL volume, CL volume, WM volume, cortical thickness, and C2–C3 CSA as candidate predictors (all with VIF < 3 to rule out multicollinearity). Among all the variables, the CL volume emerged as the first independent predictor of PIRA (OR = 1.0006 per mm³ increase, 95% CI 1.0002–1.0010, *P* = 0.005). When brain WM volume (OR = 0.89 × 10⁻¹⁵, 95% CI 0.43 × 10⁻²⁷—0.002, *P* = 0.017) entered the model at the second step, CL volume remained a significant independent predictor (OR = 1.004, 95% CI 1.002–1.008, *P* = 0.038). The ROC analysis (Fig. 4) showed that baseline CL volume predicted 4-year PIRA with an AUC of 0.691. A threshold of 403 mm³ yielded a sensitivity of 70% and specificity of 50% (PIRA prevalence 24%; positive likelihood ratio 1.4; negative likelihood ratio 0.6; post-test probability 31% versus 16%), suggesting that patients with CL volumes above this cut-off had approximately one-third probability of developing PIRA over 4 years, independently of other variables included in the model. The potential added value of combining CL and PRL loads for predicting PIRA was also explored. The combined model yielded an AUC of 0.709 compared with 0.691 for CL volume alone and 0.681 for PRL volume alone, suggesting that adding PRLs to CLs provides a slight improvement in predictive performance, although the ROC curves showed substantial overlap (Fig. 4).

## Discussion

In this 7T and 3T MRI study in a cohort of individuals with MS, we evaluated the contribution of SC and brain imaging biomarkers to baseline clinical disability and PIRA. We found that cervical SC atrophy is a major indicator of the present EDSS score, but plays a limited role in predicting PIRA compared to brain biomarkers. The contribution of WM lesions depends on the presence of smoldering activity, with PRLs providing information on the future disability trajectory and non-rim WM lesions reflecting baseline EDSS. Brain WM atrophy emerged as a strong index of current disability and risk of PIRA. Finally, we found that cortical lesion load, as assessed by 7T MRI, is the most consistent and independent driver of PIRA over a 4-year period, as well as showing an association with the current EDSS. Integrating brain and SC metrics, our multiparametric approach provides a comprehensive framework for prioritizing biomarkers with the highest clinical and prognostic relevance.

Spinal cord degeneration is widely recognized as a valid predictor of physical disability and disease progression in MS.^22,23^ A recent study identified baseline C2–C3 CSA as an independent predictor of time to PIRA in a cohort of 445 MS patients clinically followed over 4 years.^5^ In contrast, although patients who experienced PIRA in our cohort tended to have lower mean cervical SC CSA values compared to clinically stable individuals, baseline C2–C3 CSA did not significantly predict PIRA, possibly due to limited statistical power from our relatively smaller sample size. Nonetheless, our integrated analysis of brain and spinal cord measures revealed strong associations between PIRA and brain biomarkers, suggesting a predominant role of brain pathology, rather than SC atrophy, in its prediction. However, a combined assessment of brain and SC atrophy may further improve PIRA prediction. Recent work has demonstrated the feasibility of brain and cervical cord-matched quantitative 3T MRI, which also captures microstructural damage in normal-appearing WM and has shown strong predictive value for disease disability.^24^

Brain non-rim WM lesions represent the majority of the MS lesions, although disability can progress regardless of their accumulation.^25^ This suggests that other types of tissue damage may be key drivers of disease progression. Accordingly, we found that baseline non-rim WM lesion volume was primarily associated with current EDSS scores but did not demonstrate a significant predictive value for future disability. In contrast, PRLs represent a manifestation of smoldering MS, characterized by persistent inflammation at the borders of MS plaques with a rim of iron-rich active microglia/macrophages, and have been associated with a more severe disease course and disability accumulation.^12,26^ Indeed, our data revealed that baseline PRL volume is a robust predictor of the 4-year PIRA. Being associated with disability progression but not with baseline EDSS, PRLs clearly differentiate their prognostic significance from that of non-rim WM lesions. It is conceivable that PRLs impact disability accumulation as long as their intrinsic smoldering activity persists. Longitudinal quantitative susceptibility mapping (QSM) studies have shown that QSM value in the chronic active rim gradually decreases after the fourth year of their formation,^27^ and PRLs might eventually convert into non-rim WM lesions, as part of a continuum. At that point, their prognostic contribution might remain limited to the overall EDSS score, but not necessarily to PIRA.

Interestingly, we found that PRL and CL volumes share an association with disability progression, with the latter having the highest and independent impact on PIRA. There is evidence that CL volume increases significantly over time in individuals with ≥3 PRLs,^28^ who might represent patients with a more aggressive disease progression and both cortical and WM inflammatory profiles. Cortical MS lesions are strongly associated with disability progression, and in some studies, they are even more predictive than WM lesions, particularly when assessed using ultra-high field MRI.^6,28,29^ While the detection of leukocortical lesions at 3T MRI is comparable to that at 7T, the contribution of ultra-high field imaging to the identification of intracortical and subpial lesions is substantial, revealing up to ten times more lesions than 3T images.^30^ Histopathological studies have revealed that meningeal inflammation, particularly in the form of lymphoid-like structures with B-cell clonal expansion and Epstein–Barr virus (EBV)-reactivated B cells, is strongly associated with cortical pathology.^31,32^ These ectopic lymphoid follicles are predominantly observed in progressive MS and have been linked to widespread subpial grey matter demyelination. Thus, the link between CLs and PIRA may lie in compartmentalized meningeal inflammation that sustains cortical demyelination. The detection of CLs may therefore provide indirect but clinically meaningful insights into both cortical and meningeal immune activity underlying PIRA. Accordingly, although the predictive accuracy of CL volume was moderate (AUC = 0.691) and should be interpreted in conjunction with other clinical and imaging predictors, our findings suggest that CL load is the most consistent and independent marker of PIRA. However, combining CL burden with other predictive biomarkers remains the most informative approach, as demonstrated by the modest improvement in PIRA prediction when PRL and CL loads were combined (AUC = 0.709).

Our study also confirmed that brain WM atrophy is highly associated with both current disability and PIRA.^6,33^ In contrast, cervical SC CSA was not retained as a significant predictor of PIRA. However, brain and SC volumetry has become a valuable tool for understanding MS progression, reflecting the neuroaxonal loss occurring in the CNS. This may represent the effect of subtle chronic inflammation that persists throughout the disease course, leading to greater atrophy in patients with more pronounced smoldering activity. In particular, brain WM may be more closely associated with compartmentalized inflammation, given its proximity to key sources of such inflammation, including enlarged choroid plexuses,^34^ PRLs,^35^ and diffuse microglial activation within the normal-appearing WM.^36^

The present study builds upon our previous machine learning work,^6^ which identified PRLs, leukocortical lesion volumes and brain WM volume as principal predictors of EDSS progression. Here, we: (i) assessed SC atrophy as an additional, albeit minor contributor to clinical progression; (ii) refined the clinical outcome by isolating PIRA, thus capturing the clinical manifestation of smoldering MS; (iii) increased the sample size (from 71 to 97 patients followed longitudinally) and the clinical follow-up period (from a mean of 3 to 4 years); and (iv) validated our findings using conventional statistical approaches, thereby reinforcing the robustness of the observed associations.

This work has some limitations. A critical factor to consider when interpreting our results is that we did not assess the contribution of SC lesions to neurologic disability. However, a previous study with 3-year clinical follow-up did not show any correlation between baseline SC lesion number and change in disability measures, including EDSS, 25-foot walk test, 9-hole peg test, Paced Auditory Serial Addition Test and Symbol Digit Modalities Test,^28^ suggesting a limited contribution of SC lesions compared with SC atrophy.^5^ Moreover, we did not evaluate other established MRI biomarkers of PIRA, such as slowly expanding lesions (SELs) and choroid plexus enlargement. Although SELs represent another form of chronic active lesion, they show only around 20% spatial overlap with PRLs, meaning that we may have missed a proportion of aggressive WM lesions relevant to progression.^9,12,37,38^ Our findings should also be integrated with choroid plexus volume, as choroid plexus enlargement, regardless of disease stage, has been associated with non-rim and PRL burden, and increased risk of PIRA.^39,40^ Another limitation is that clinical phenotype and disease duration, despite their known relevance to MS disability, were not included as covariates in the multivariable models. When tested, they strongly predicted EDSS and PIRA, limiting our ability to assess the independent predictive value of imaging markers. However, they were included in the stepwise logistic regression model, but neither was retained in the models. The exploratory analyses we performed, stratified by clinical phenotype, yielded consistent results, supporting CLs and WM atrophy as key predictors throughout the disease course. Finally, follow-up duration varied across patients in our cohort, with a mean of 4 years. Although follow-up time was included as a covariate, this heterogeneity may have influenced the detection of PIRA events, which are inherently time-dependent.

In conclusion, our 7T and 3T MRI study maps the clinical trajectory of numerous SC and brain biomarkers of disability in patients with MS. The CL load has proven to be the strongest independent predictor of PIRA within 4 years, underscoring the necessity for new imaging techniques able to increase the sensitivity for CLs in the clinical setting.

## Supplementary Material

fcag059_Supplementary_Data

## Data Availability

The data sharing depends on Massachusetts General Hospital and its Institutional Review Board policy, as well as on the purpose of sharing the data (profit versus nonprofit).
