# Supplementary material for: Spinal cord versus brain imaging biomarkers of multiple sclerosis trajectory combining 7T and 3T MRI
Source: Brain Commun. 2026 Feb 25;8(2):fcag059. doi: 10.1093/braincomms/fcag059 (PMC13036496; doi:10.1093/braincomms/fcag059)
Supplement: fcag059_Supplementary_Data [file fcag059_supplementary_data.pdf]

Supplementary material

Supplementary Table 1. MRI protocol

Field Strength	Sequence	Acquisition plane	3D vs 2D	TR (ms)	TE (ms)	TI (ms)	FA (°)	Voxel size (mm)	Slice number	FOV (mm, AP x RL x SI)
7T	T2*-weighted FLASH GRE	Axial	2D	1700	21.8	-	55	0.33x0.33x1	80 (2 slabs)	210x210x80
7T	T1-weighted MPRAGE	Axial	3D	2600	3.26	1100	9	0.6x0.6x1.5	96	194x192x144
3T	T1-weighted MEMPRAGE	Sagittal	3D	2530	1.7, 3.6, 5.4, 7.3	1200	7	0.9x0.9x0.9	208	230x230x208

Siemens 7T and 3T MR scanners using a 32-channel head coil

Abbreviations: TR: repetition time. TI: inversion time. TE: echo time. FA: flip angle. FOV: field of view. AP: anterior-posterior. RL: right-left. SI: superior-inferior. FLASH GRE: fast low-angle shot gradient echo. MPRAGE: Magnetization Prepared Rapid Gradient Echo. MEMPRAGE: multi-echo magnetization-prepared rapid gradient echo.

Supplementary Table 2. Correlations between MRI biomarkers and disability measures stratified by clinical phenotype

Spearman's Correlations								
	RRMS				SPMS			
	Baseline EDSS		PIRA		Baseline EDSS		PIRA	
MRI variables	Rho	p-value	Rho	p-value	Rho	p-value	Rho	p-value
Non-rim WM lesion volume	0.192	0.083	0.021	0.860	0.376	0.053	0.396	0.054
PRL volume	0.146	0.189	0.143	0.224	0.163	0.416	0.375	0.077
CL volume	0.303	**0.006**	0.060	0.616	0.122	0.544	0.502	**0.015**
Brain WM volume	-0.105	0.346	-0.317	**0.006**	-0.333	0.090	-0.317	0.140
Cortical Thickness	-0.176	0.111	-0.185	0.114	-0.077	0.703	-0.119	0.589
C2-C3 CSA	-0.108	0.335	-0.115	0.337	-0.291	0.190	-0.151	0.550

Abbreviations: CL: cortical lesion; CSA: cross-sectional area; EDSS: Expanded Disability Status Scale; PIRA: Progression independent of relapse activity; PRL: paramagnetic rim lesion; RRMS: relapsing-remitting MS; SPMS: secondary progressive MS; WM: white matter
